# Temperature and Pressure Wireless Ceramic Sensor (Distance = 0.5 Meter) for Extreme Environment Applications

**DOI:** 10.3390/s21196648

**Published:** 2021-10-06

**Authors:** Justin Daniel, Spencer Nguyen, Md Atiqur Rahman Chowdhury, Shaofan Xu, Chengying Xu

**Affiliations:** 1 Department of Mechanical Engineering, Florida State University, Tallahassee, FL 32306, USA; jbd12b@my.fsu.edu (J.D.); snguyen95@gmail.com (S.N.); 2 Mechanical and Aerospace Engineering Department, North Carolina State University, Raleigh, NC 27695, USA; chowdhury.r.atique@gmail.com (M.A.R.C.); sxu27@ncsu.edu (S.X.)

**Keywords:** extreme environments, passive wireless sensors, patch antenna design, polymer-derived ceramic, radio frequency identification, temperature sensor, pressure sensor

## Abstract

This paper presents a design for temperature and pressure wireless sensors made of polymer-derived ceramics for extreme environment applications. The wireless sensors were designed and fabricated with conductive carbon paste on an 18.24 mm diameter with 2.4 mm thickness polymer-derived ceramic silicon carbon nitride (PDC-SiCN) disk substrate for the temperature sensor and an 18 × 18 × 2.6 mm silicon carbide ceramic substrate for the pressure sensor. In the experiment, a horn antenna interrogated the patch antenna sensor on a standard muffle furnace and a Shimadzu AGS-J universal test machine (UTM) at a wireless sensing distance of 0.5 m. The monotonic relationship between the dielectric constant of the ceramic substrate and ambient temperature is the fundamental principle for wireless temperature sensing. The temperature measurement has been demonstrated from 600 °C to 900 °C. The result closely matches the thermocouple measurement with a mean absolute difference of 2.63 °C. For the pressure sensor, the patch antenna was designed to resonate at 4.7 GHz at the no-loading case. The sensing mechanism is based on the piezo-dielectric property of the silicon carbon nitride. The developed temperature/pressure sensing system provides a feasible solution for wireless measurement for extreme environment applications.

## 1. Introduction

Wireless passive sensors for ultra-harsh conditions are greatly needed to help with structural integrity, health monitoring, and proximate environment monitoring. Compared to physical wires, wireless devices are relatively inexpensive to maintain in a high-temperature environment and are less susceptible to failure [1]. Existing methods for remote sensing (i.e., passive inductor and capacitor resonant telemetry scenes, accelerometer, surface acoustic wave sensor, chemical resistor, etc.) are performed by proximate environmental monitoring and are primarily limited to storage and transportation purposes; they are usually limited by sensing distance (e.g., ≤3 cm) [2]. However, the development of next-generation defense systems demands greater situational awareness of the extreme environmental conditions (i.e., acceleration, temperature, pressure). Therefore, novel sensors that can increase the safety by tracking and assessing their status on demand will find great utility in many defense applications. This kind of device can keep the effectiveness of material over extended periods of time under extreme and transient environment conditions. It is envisioned that traditional sensors can be improved upon by utilizing novel materials (formulated for extreme pressure, temperature, and vibration environments) in combination with additive manufacturing (AM) techniques to work towards true 3D sensing capability. Additionally, it is desirable to produce ‘shock hardened’ electronics by scale-tailorable AM packaging for fuse electronics, which has the ability to miniaturize manufactured devices.

This study demonstrated wireless patch antenna temperature and pressure sensors that operate at a greater sensing distance improved upon by utilizing novel materials (formulated for extreme temperature and vibration environments). The patch antenna temperature and pressure sensors consist of a polymer-derived SiCN (silicon carbon nitride) ceramic substrate, a carbon paste patch, and a ground plane. Since the patch is a resonating element, the patch antenna sensor receives interrogation signals at its resonant frequency. The resonance of the patch is coupled to the dielectric constant of the substrate, which changes with the variation of temperature and pressure [3]. Therefore, the temperature and pressure measurements performed are wireless, using the resonant frequency signal. To identify resonant frequency changes, the S_11_ parameter is measured. While a patch antenna has been used as a wireless sensor node in previous research, the proposed patch sensor operates at a greater sensing distance compared to other reports in the existing literature [4,5,6,7].

The embedded sensor developed here can be additively manufactured with novel ruggedized material into complex 3D geometries, which is able to realize 3D sensing in the high temperature/shock environment of munitions (up to 50 MPa). In the literature, the majority of existing additively manufactured wireless sensors are made on extruded polymer substrates which have negligible resistivity change and no delamination for temperature cycling from room temperature to as high as 300 °C [8,9,10,11,12]. Above this temperature range, the antenna (or substrate) would not survive, or delamination/cracking will occur which prevents the sensor from functioning correctly.

The benefits of using additive manufacturing (AM) in this research include the potential to directly print the sensor onto conformal substrates as well as embedding the sensor into the testing materials with complex geometries. Additionally, the form factor and design can be rapidly prototyped with AM for proof of concept. This research work optimizes the materials and design for embedded temperature and pressure measurements while simultaneously testing the sensor performance to develop a robust, embedded sensor.

In this paper, the PDC temperature and pressure sensors were designed, fabricated, and experimentally demonstrated in high-temperature settings from 600 °C to 900 °C and in force from 0 N to 4500 N. Section 2 presents the fabrication method and sensing material property of the PDC which enables it to sense the variations in temperature and pressure. In Section 3, the operation principle and geometry design of a micro-patch antenna sensor based on the transmission line theory as well as the reflection are described. The experimental setup, digital signal processing procedure, and experimental results are explained in Section 4. Finally, the summary and conclusion are presented in Section 5.

## 2. Sensing Material and Property Characterization

### 2.1. PDC-SiCN Material Property

**Figure 1:**
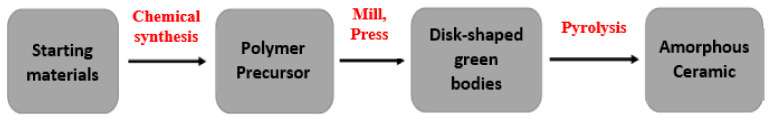
Processing steps for synthesizing PDC (SiCN) material.

The wireless sensing material was made of the polymer-derived silicon carbonitride (SiCN) ceramic and was fabricated using commercially available polysilazane (Kion Ceraset, USA) as the precursor. Polysilazane is a polymer consisting of nitrogen and silicon atoms alternating in their backbone. For the preparation, 8.8 g liquid-phased Polysilazane and 1 g aluminum-tri-sec-butoxide were mixed at 120 °C for 24 h while 2 wt% of the diluted catalyst solution was slowly added to the mixture. Curing took place at 140 °C and 350 °C, then the crosslinked samples were ball-milled (8000D Mixer/Mill^®^, SPEX SamplePrep, Metuchen, NJ, USA) before pressing and being put into a tube furnace (carbolite gero 30–3000) for pyrolysis. The samples were heated up to 1000 °C. The detailed processing steps can be seen in Figure 1.

Based on Figure 1, the process of SiCN substrate fabrication is: First, the liquid polysilazane is solidified with 2 wt% of dicumyl peroxide (DP) as the thermal initiator and subsequently cross-linked respectively at 140 °C and 350 °C for 3 h in nitrogen. The cross-linked product is then ground for 90 min to a fine powder of about 10 μm using a planetary ball mill. During grinding preparation, five drops of liquid polysilazane are added for every 1 g (of) stirred fine powder, and the product is mixed with 3 g of 140 °C thermal treatment product and 3 g of 350 °C thermal treatment product in a mortar. Under a uniaxial pressure of 4.83 MPa, the powder is compressed into disk samples of 18.24 mm in diameter and 0.5–1.0 mm in thickness. Finally, the compacts are pyrolyzed at 1000 °C for 3 h.

**Figure 2:**
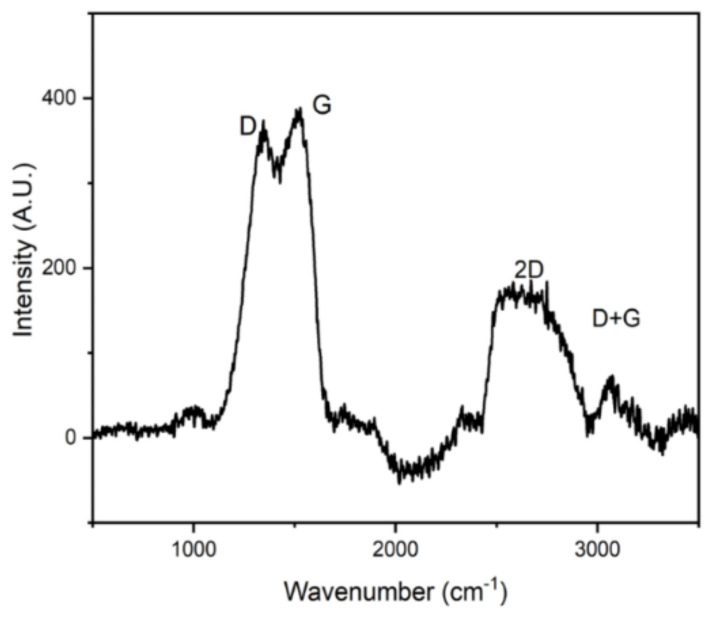
Micro-Raman spectroscopy measurements of the polymer-derived SiCN.

Micro-Raman spectroscopy investigations were performed for the as-prepared SiCN sensor with the pyrolysis of 1000 °C, as shown in Figure 2. The recorded spectra show the presence of typical bands for disordered sp^2^ carbon. The two high-intensity Raman features of the segregated carbon phase in PDC are the so-called D and G bands at 1340 cm^−1^ and 1600 cm^−1^. The G band is related to the stretching mode of the sp^2^ hybridized carbon atoms in rings and/or chains whereas the D band relies on breathing modes of the rings and occurs only in disordered ring structures. Moreover, two bands at around 2690 and 2920 cm^−1^ are attributed to overtones or combination modes of the D- and G- bands. Free carbon plays an important role in the dielectric behavior of the PDC systems.

**Figure 3:**
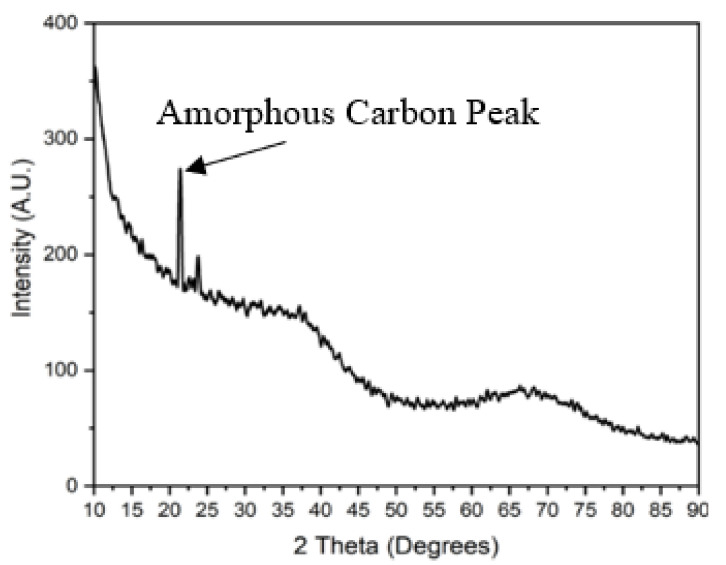
XRD patterns of PDC-SiCN ceramics at 1000 °C.

**Figure 4:**
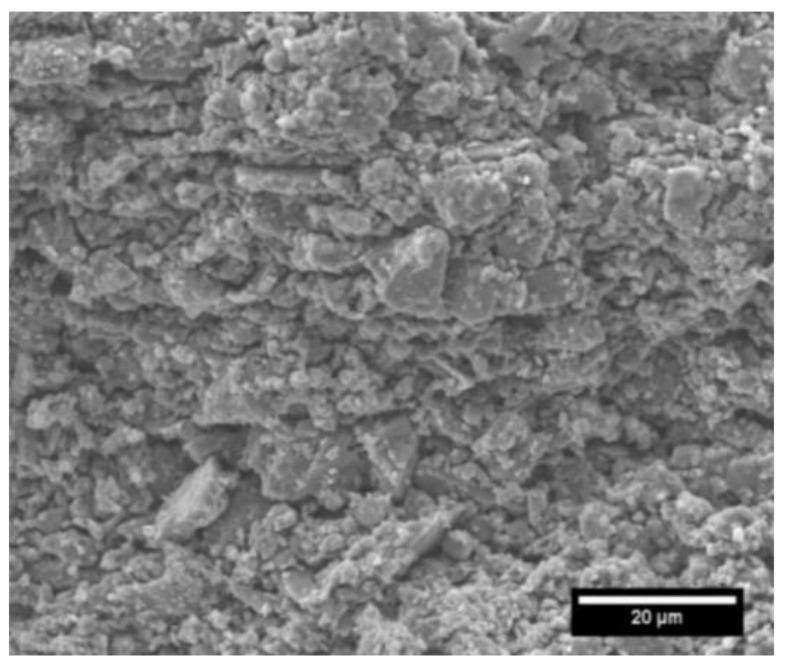
SEM image of PDC-SiCN pyrolyzed at 1000 °C showing the amorphous nature of the matrix.

In order to investigate the microstructure, XRD (ICDD PDF Card NO.33-1160) and SEM were carried out. Figure 3 shows the XRD patterns of SiCN ceramics pyrolyzed at 1000 °C. The diffraction peak around 20° belonged to amorphous carbon. The spectrum indicates that the microstructure is amorphous and devoid of crystalline SiC. The presence of the carbon peak confirms the influence of the carbon in the structure. The SEM image in Figure 4 also demonstrates that the PDC-SiCN matrix is amorphous which is in agreement with our previous findings [13,14].

### 2.2. Fabrication of the PDC-SiCN Sensor

**Figure 5:**
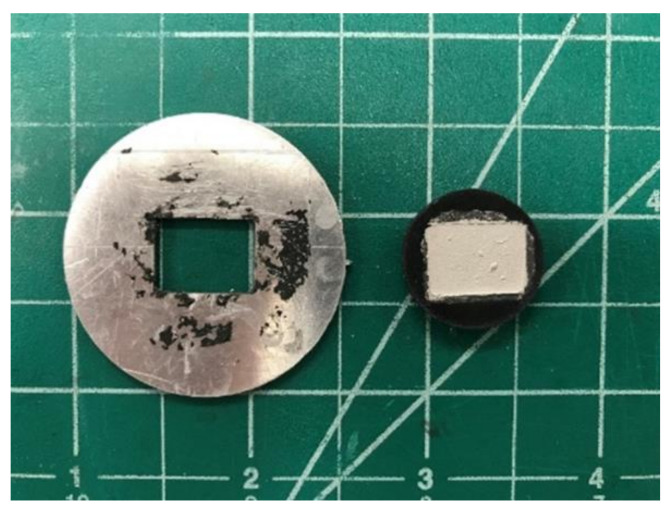
MPA mold (**left**) and PDC substrate (**right**) with conductive trace applied.

When making the wireless PDC-SiCN sensor, the surface of specimens was firstly polished before coating with the carbon paste. Then, a layer of carbon paste (Pelco, Redding, CA, USA) from an aqueous solution was applied on both the top (seen in Figure 5, right) and bottom surfaces of the substrate to form the rectangular patch and ground plane. An inverse mold is made by machining the dimensions of the patch out of a flat material (seen in Figure 5, left). The conductive paste is then applied until the patch dimensions and the ground plane side of the substrate are coated. The substrate and conductive traces are then cured using a heat treatment process that bonds the paste to the substrate and increases the conductivity of the trace.

**Figure 6:**
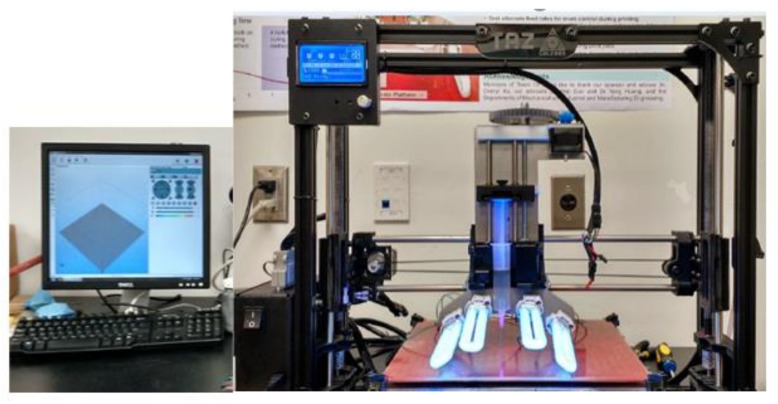
3D-printing experimental setup.

**Figure 7:**
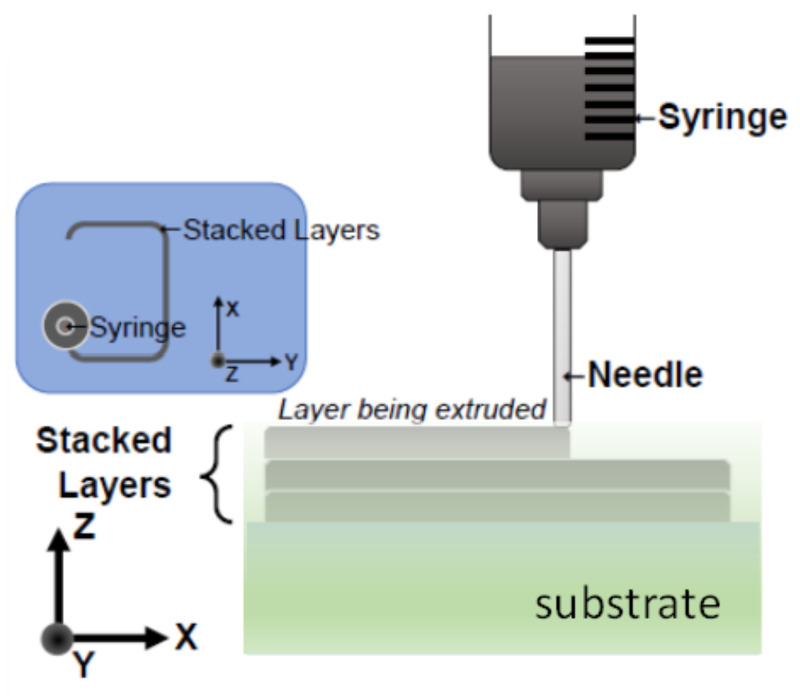
Schematic diagram of antenna 3D printing process.

With PDC-SiCN as the substrate and carbon paste as the conductive trace, the sensor can be fabricated by the 3D printing technique. The 3D-printing experimental setup is shown in Figure 6. Material is stored in a syringe and sprayed by the printing needle. The antenna is printed layer by layer as an embedded structure in the substrate as shown in Figure 7.

### 2.3. Monotonic Relationship between the Dielectric Constant of Sensor Substrate and Ambient Temperature/Applied Pressure

**Figure 8:**
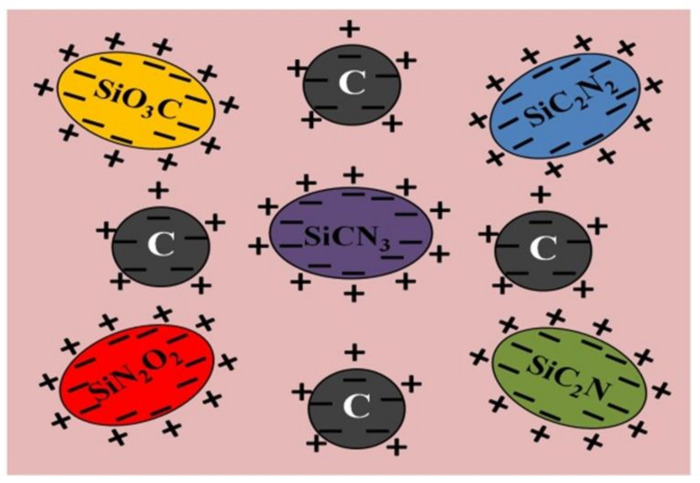
Schematic diagram of space charge polarization of PDC-SiCN when an alternating electromagnetic field is applied.

The amorphous structure of SiCN contains various chemical phases including SiCO_3_, SiN_2_O_2_, SiCN_3_, SiC_2_N_2_, and SiCN_3_ [15]. In these units, there are many Si- and C-related dangling bonds. The amorphous matrix phase consists of various chemical units and highly disordered carbon. The dielectric property of PDC-SiCN is closely related to its structure. The dielectric constant is mainly ascribed to the space charge polarization in which the ions and electrons are activated and moved to the interface of free carbon phases. The space charge polarization depends upon the number of ions and electrons near these structures, as illustrated in Figure 8.

#### 2.3.1. Wireless Temperature Sensing Principle

At low temperatures, the energy for the thermal motion of these particles is low. The relaxation polarization is difficult to initiate which results in a long relaxation time. In this case, the motion of the relaxation particles cannot keep pace with the change of the applied electric field. The dielectric constant of the PDC-SiCN, therefore, is low. With an increase in ambient temperature, the relaxation particles acquire more energy due to the thermal motion, which results in the decrease in relaxation time. This change can be described in (1) [16].

(1) $$\tau \left(T\right)=\tau_{0}\exp(\frac{E_{a}}{R\cdot T})$$

where *T* is temperature, *τ*_0_ is a pre-factor, *E_a_* is the activation energy, *R* is gas constant, and *τ*(*T*) is the temperature-dependent polarization relaxation time. It is clear that *τ*(*T*) decreases as temperature increases, and polarization transformation occurs easily in the electric field. The motion of the relaxation particles can keep pace with the change of electric field and the setup of relaxation polarization is easier. Thus, the dielectric constant of the PDC-SiCN increases when the temperature increases, as shown in (2) [16].

(2) $$\varepsilon^{\prime }=\varepsilon_{\alpha }+\frac{\varepsilon_{s}-\varepsilon_{\alpha }}{1+\omega^{2}\cdot \tau {\left(T\right)}^{2}}$$

where *ε_s_* is static dielectric constant, $\varepsilon_{\alpha }$ is relative dielectric constant at a high-frequency limit, and *ω* is the angular frequency. The relaxation polarization becomes prominent at elevated temperatures [17]. Thus, the dielectric constant of the PDC sensor substrate has a monotonic relationship with the ambient temperature.

#### 2.3.2. Wireless Pressure Sensing Principle

The sensing mechanism in this wireless RF pressure sensor design is based on the piezo-dielectric and piezo-resistivity property of PDC [4,18]. As a mechanical load is applied to the sensor, the dielectric constant of the PDC changes. To explain, the pressure coefficient for dielectric constant is introduced as shown in (3):

(3) $$\kappa =\frac{d\varepsilon /\varepsilon }{dp}=\frac{\partial \ln\varepsilon }{\partial p}$$

where ε is the dielectric constant and *p* is the pressure. The relationship between dielectric constant and polarizability is the famous Clausius-Mossotti (CM) relationship, as shown in (4) [19].

(4) $$\frac{M}{\rho }\left(\frac{\varepsilon -1}{\varepsilon +2}\right)=\frac{4\pi }{3}N\alpha$$

where M is the atomic weight in grams, ρ is the density, N is Avogadro’s number, α is the atomic polarizability and ε is the dielectric constant. By simplifying (4) and substituting it into (3), the new relationship is shown in (5) [19].

(5) $$\kappa =\frac{\partial \ln\varepsilon }{\partial p}=\frac{\left(\varepsilon -1\right)\left(\varepsilon +2\right)}{3\varepsilon }\left(\frac{\partial \ln\alpha }{\partial p}+\beta \right)$$

where β is the part related to the compressibility of materials and can be neglected because it is much smaller compared with the first part in brackets. Thus, the increase in dielectric constant with pressure has attributed to an increase in polarization. The change of polarization caused by the increase in pressure can be demonstrated as (6) [20] by rearranging Equation (5).

(6) $$\frac{\partial \ln\alpha }{\partial p}=\frac{3}{\varepsilon }\frac{\partial \ln\varepsilon }{\partial p}$$

According to the PDC material’s property, $\frac{\partial \ln\alpha }{\partial p}$ is consistently positive which indicates that polarization is increasing with increasing pressure. Thus, the dielectric constant of the PDC sensor substrate has a monotonic relationship with the applied pressure.

## 3. Micro-Patch Antenna Theory

### 3.1. Operation Principle of the Wireless Patch Antenna

**Figure 9:**
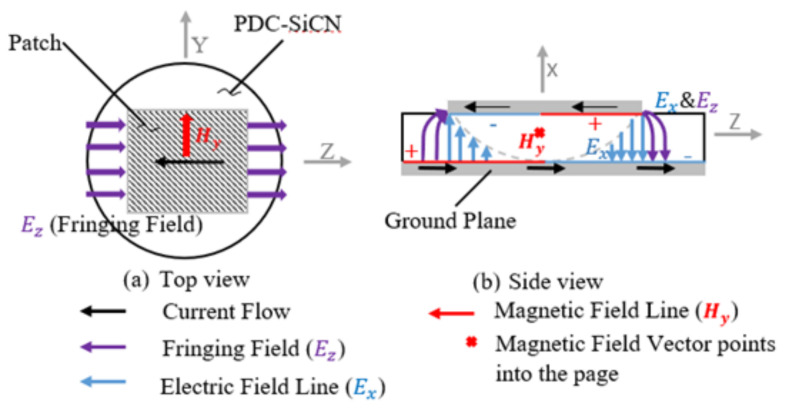
Electric and magnetic fields of the PDC patch antenna sensor.

The wireless interrogation system is composed of a horn antenna, a patch antenna sensor, and a vector network analyzer which performs frequency sweeps and signal measurements. The antenna radiating elements consist of a metallization applied on the top (patch) and the bottom (ground plane) of the sensor as shown in Figure 9. The presented temperature and pressure sensor enables the patch antenna for wireless operations. The sensor substrate material, PDC-SiCN, is selected since it has good high-temperature mechanical stability [21] and dielectric properties [22]. This simple structure of the patch antenna sensor is desirable because it is lightweight, has a low profile, and is inexpensive to fabricate [23].

The sensor operation begins with the horn antenna broadcasting frequency sweeps toward the patch antenna sensor. The frequency component transmitted to the patch antenna sensor can be introduced and stored inside the sensor (substrate) or reflected back to the horn antenna. The interrogation frequency close to the resonant frequency of the sensor establishes the desired power transfer to the sensor due to the minimum impedance at its resonance. The power coupling induces the surface charges on the inside of conducting plates and produces electric fields between two separate conductors. In addition, the current flow in the patch and ground plane creates magnetic fields. The formation of magnetic fields results in electromagnetic energy being trapped inside the sensor and operating as a transmission line model as shown in Figure 9. However, in the case of the interrogation signal differing from the resonant frequency of the sensor, the electromagnetic energy rather reflects the horn antenna because the impedance blocks the power passing through the sensor [24].

The signal measurement of the reflection and absorption response is distinctive in the return loss (S_11_); a definitive trough appears at its resonant frequency. Thus, due to the ease of reading the resonant frequency of the sensor, it is reliable as a sensing signal. The dielectric constant is an important element in deciding the resonant frequency of the sensor such that its change (due to an increase in temperature and pressure) is attributed to the change in the resonant frequency of the sensor. Therefore, the temperature and pressure can be measured wirelessly using the resonant frequency of the sensor.

### 3.2. Wireless Measurement Distance

The electromagnetic power transfer between the interrogator and receiver is relevant in order to achieve the wireless operation of the sensor. The wireless power transfer performance between a transmitter and receiver can be approximated by the Friis transmission equation as shown in (7) [25].

(7) $$P_{r}=\frac{A_{r}\cdot A_{t}}{d^{2}\cdot \lambda^{2}}\cdot P_{t}$$

where *P_t_* is the power fed into the transmitting antenna, *P_r_* is the power available at the receiver, *A_t_* is the effective area of the transmitting antenna, *A_r_* is the effective area of the receiving antenna, *d* is the distance between antennas, and λ is the wavelength. The Friis transmission equation indicates that when the receiver sensor size and transmitted power are fixed, power transfer performance depends on the aperture size of the transmitter and the distance between the two antennas.

Since a horn antenna is well-known to provide a directional radiation pattern and a large gain [26], it can deliver strong energy to the sensor. In addition, the large aperture size of 27.95 cm × 19.05 cm (11 in × 7.5 in) provides a large effective transmitting area for wireless power transmission. Thus, a horn antenna is selected as an interrogator in the wireless operation of the patch antenna sensor; despite the small effective area of the sensor, it still can fulfill a long interrogation distance. In addition to power transmission from the interrogator, since the reflected return is a sensing signal as well, the reflection intensity degradation by the square of the distance is also considered. The interrogation distance was adjusted through repeated experiments in the near radiating field.

In the experiment, the distance of 0.5 m (20 inches) from the sensor to the horn antenna is determined through experiments that provided repeatable signals in several experimental runs. The distance choice is also corresponding to the far-field region, which is defined as (8).

(8) $$2D^{2}/\lambda \leq R$$

where *R* is the boundary distance, *D* is the largest dimension of the antenna, *λ* is the wavelength of the frequency [25].

### 3.3. Geometry Design of Patch Antenna

The design of the patch antenna sensor resonating at a specific frequency is essential for a wireless sensor since the resonant frequency of the sensor is dependent upon the dielectric constant. The variation of temperature and pressure will change the dielectric constant of the substrate. In other words, the detection of its resonant frequency is crucial to measure the temperature and pressure. The resonant frequency of the sensor is a reliable sensing signal due to the phenomenon observed only at resonance. When electromagnetic waves transmit at the same frequency with the resonant frequency of the sensor, it is capable of propagating at the largest amplitude through the sensor. This resonance encourages minimization of sensor impedance which allows it to maximize signal transfer with minimal loss.

The resonant frequency of the patch antenna sensor is determined based on the dielectric constant of the dielectric substrate and the geometry of the patch antenna sensor. The material dielectric constant is a complex value that is presented in (9) [27].

(9) $$\varepsilon =\varepsilon^{\prime }-j\varepsilon^{\prime \prime }$$

where $\varepsilon^{\prime }$ is the real part of the dielectric constant and $\varepsilon^{\prime \prime }$ is the imaginary part of the dielectric constant. The real part of the dielectric constant represents the electric energy retained in material from an external electric field, and the imaginary part of the dielectric constant represents the loss of electric energy to an external electric field.

In terms of the dielectric constant, the resonance phenomenon is governed by the real part since it allows most of the power to transfer into the sensor and to be stored in the material with significantly less loss. The imaginary part of the dielectric constant for SiCN is significantly small in a span of temperature range, e.g., tan δ ≈ 0.023 at 500 °C [28]. Because of this small value, it is reasonable to only take into account the real part of the dielectric constant in determining its resonant frequency.

The geometrical design of the patch and the height of the substrate is significant in constructing the field configuration beneath the patch at its resonant frequency. The patch antenna sensor exhibits that the electric field lines pass through non-homogeneous media, typically air, and a dielectric substrate because the dimensions of the patch are finite along the length and width as shown in Figure 8. For this reason, the electric field has different phase velocities in the two media, so it undergoes fringing at the edges of the patch (E_z_ in Figure 9). Therefore, the patch antenna sensor can be described as two radiating slots separated by a transmission line [29]. The fringing field is taken into account for the design of the sensor.

The resonant frequencies of the patch antenna temperature and pressure sensor were initially designed at about 5.75 GHz and 4.7 GHz, respectively. The geometry of the patch antenna sensor is approximated with the following steps.

The patch width W can be calculated using (10) [23].

(10) $$W=\frac{C}{2\cdot f_{r}}\sqrt{\frac{2}{\varepsilon_{r}+1}}$$

where C is the speed of light, $f_{r}$ is the designed resonant frequency, $\varepsilon_{r}$ is the effective dielectric constant.

The substrate thickness h is suggested to be a small fraction of a wavelength (0.003*λ*_0_ < *h* < 0.05*λ*_0_, where *λ*_0_ is the free-space wavelength) [23]. In general, as the substrate thickness increases, the number of fringing increases. The fringing field is the reason for the patch antenna sensor to radiate.

The effective dielectric constant $\varepsilon_{reff}$ takes into account the fringing effect so that the nonhomogeneous electrical characteristics in air and the substrate are considered as a uniform dielectric in the patch design. An effective dielectric constant represents the mixed dielectric constant of the substrate and air, which can be estimated by (11) [23].

(11) $$\varepsilon_{reff}=\frac{\varepsilon_{r}+1}{2}+\frac{\varepsilon_{r}-1}{2}{\left[1+12\frac{h}{W}\right]}^{-\frac{1}{2}}$$

where $\varepsilon_{r}$ is the relative dielectric constant, *h* is the substrate thickness and *W* is patch width.

Fringing makes the patch appear larger in the electrical field plane to be extended on each side of the patch by a distance ∆*L*, which is shown in (12) [23].

(12) $$∆L=0.412h\frac{\left(\varepsilon_{reff}+0.3\right)\left(\frac{W}{h}+0.264\right)}{\left(\varepsilon_{reff}-0.258\right)\left(\frac{W}{h}+0.8\right)}$$

where $\varepsilon_{reff}$ is the effective relative dielectric constant calculated in (11).

For a rectangular patch, the length of the patch is usually *λ*_0_/3 < *L* < *λ*_0_/2 [23]. The length of the patch should ideally be a half-wavelength. At this length, the patch creates the maximum electric field in the smallest dimensions of the patch. For the patch antenna sensor, the actual length of the patch becomes slightly less than a half-wavelength because of the fringing effect. According to the designed resonance frequency and equation (12), the effective length of the patch can be obtained in (13).

(13) $$L_{\mathrm{eff}}=L+2\Delta L=\frac{c}{2f_{r}\sqrt{\varepsilon_{\mathrm{reff}}}}=\frac{\lambda_{d}}{2}$$

where $\lambda_{d}$ is the wavelength of the designed resonance frequency and $f_{r}$ is the designed resonance frequency.

The effective length varies as the extended length is added to the actual length of the patch as shown in (13). According to the relationship between the real length and effective length of the patch, the real patch length that satisfies our design can be obtained in (14).

(14) $$L=L_{eff}-2∆L$$

## 4. Experimental Setup

### 4.1. Time-Domain Gating Principle

To obtain the resonant frequency using a wireless interrogation method, filtering techniques must be performed. As the electromagnetic waves propagate through the environment, they are reflected by physical objects that cause noise in the S_11_ reflection coefficient measurement. Since testing is done using a metal machine with high reflections, data math is performed to subtract the noise from the measurement. The horn antenna and machine are set up without the sensor in place. The data from the S11 response are saved and then subtracted from the real-time response.

In the experiment, a Digital Signal Processing (DSP) algorithm was used to extract the resonant frequency of the sensor from the measured S_11_ [30]. First, the S_11_ responses of the experiment environments including a furnace/universal test machine (UTM) and a horn antenna were measured without a patch antenna sensor. Inverse Fast Fourier Transform (IFFT) was used to transform from the frequency to the time domain. In the time domain, the S_11_ response was calibrated to characterize the structural backscattering due to reflections inside of the furnace and UTM. These internal reflections were filtered out by subtracting the reflections from the S_11_ graph. Second, the sensor was placed inside the furnace, and UTM and another calibration were performed in order to isolate the sensor.

**Figure 10:**
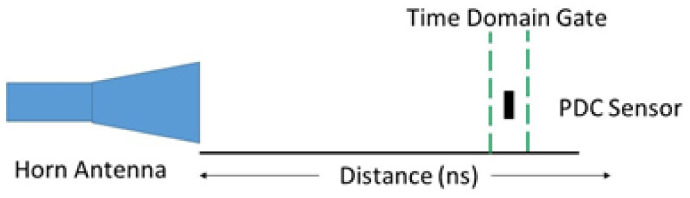
Time−domain gating filtering technique diagram of the experiment setup.

In both experiments, a time-domain technique is used to find the sensor within the wireless environment. A time of 0 s corresponds to the feeding point of the transmitting interrogation horn antenna. The reflection wave packet is maximized by placing a high reflective metal plate in the same location of the sensor, which can be found as a peak in time domain response. The location is given in nano-seconds away from the port (interrogation antenna). A gate is then applied to encompass the peak shown in Figure 10. After the time gate was applied, a Fast Fourier Transform (FFT) was used to convert back to the frequency domain from the time domain. The final process signal is then scaled, and the lowest amplitude is determined as the resonant frequency of the sensor.

**Figure 11:**
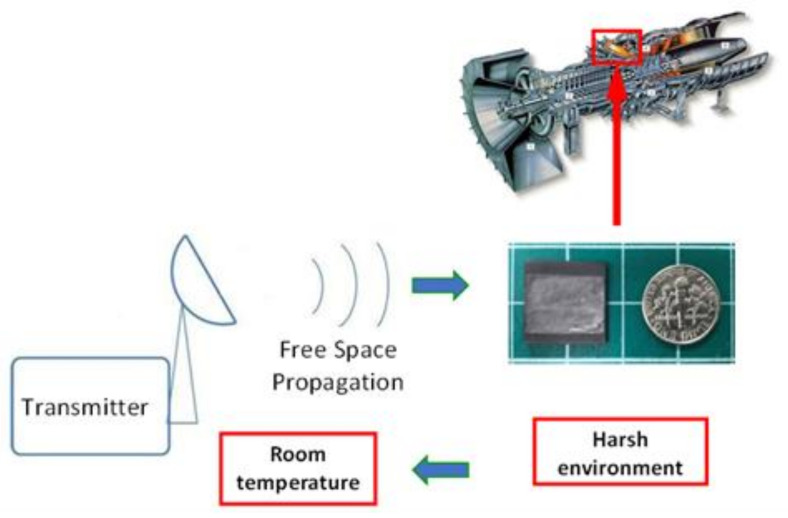
Wireless PDC temperature/pressure sensor that can be used to monitor the internal condition of a turbine engine.

A wireless sensing system with high-temperature survivability and a small volume is significant in that it can be used in extreme situations such as harsh environments over a variety of temperatures. The wireless sensing system may be utilized within the surface of simple devices to the interior of complicated mechanical structures such as turbine engines and nuclear reactors. As shown in Figure 11, a temperature/pressure sensor the size of a coin may be used to monitor the internal condition of a turbine engine.

### 4.2. Experiment Setup of Wireless Temperature Sensor

**Figure 12:**
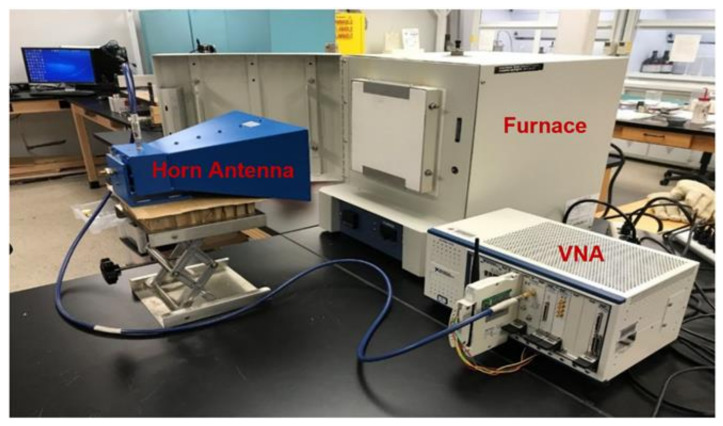
Temperature sensor experimental setup.

The experimental setup to validate the temperature sensing capability of a patch antenna sensor with a wireless interrogation is shown in Figure 12. The muffle furnace was constructed from Moldatherm Insulation. The foam box was used because it is a radio frequency transparent material and a good thermal insulator. Thus, the temperature of the furnace could increase uniformly without the metallic door in place.

The wireless interrogator was conducted by using a vector network analyzer (VNA). The VNA was a PXI-M9375a from Keysight Technologies that produced a frequency sweep 0.6–8 GHz with a gain of 6–15 dB and was broadcast by a horn antenna positioned 0.5 m away from the furnace. The signal travels through free space until it reaches the sensor. The horn antenna is connected to a one-port VNA so that the S_11_ parameter can be acquired. A k-type thermocouple was placed inside the furnace to verify the temperature. The thermocouple measurements were acquired by a PXIE- thermocouple DAQ.

### 4.3. Experiment Setup of the Wireless Pressure Sensor

**Figure 13:**
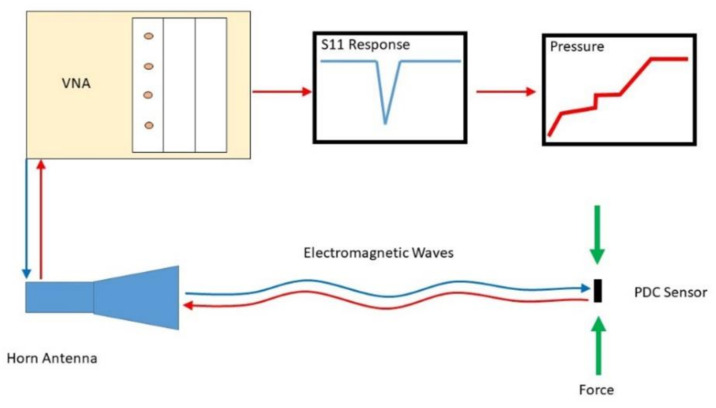
Wireless pressure sensing process diagram.

In order to test and validate the pressure sensor design, a National Instruments Vector Network Analyzer (VNA) PXIe-5630 (0.1–6 GHz) along with a wideband horn antenna (0.8–6 GHz) were used. The diagram can be seen in Figure 13. The VNA applied a frequency sweep from 3–6 GHz in 0.0015 GHz steps. The horn is placed so that the antenna pattern is normal to the surface of the patch on the sensor. The sensor was placed in a Shimadzu AGS-J universal test machine (UTM). The sensor was positioned so that the patch faced the horn. The horn antenna was placed 0.5 m away from the sensor. This distance was chosen due to the high reflective metal construction of the UTM. It is possible to increase this distance in low reflection environments or by using a different interrogation antenna with a more focused beam pattern.

**Figure 14:**
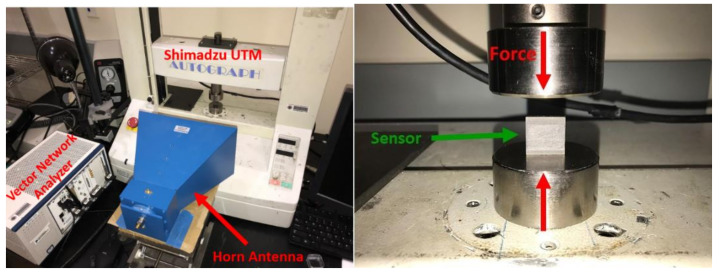
Pressure sensor experimental setup.

The sensor was placed in the UTM so that the load is applied to the thin edges of the sensor. In this orientation, the patch maintains its position normal to the horn antenna (seen in Figure 14). A 5000 N load cell was used in the UTM to validate the forces. A load was applied to the sensor in increments of 250 N. The resonance was recorded at each step until 4500 N of force was loaded on the sensor.

## 5. Result and Discussion

**Figure 15:**
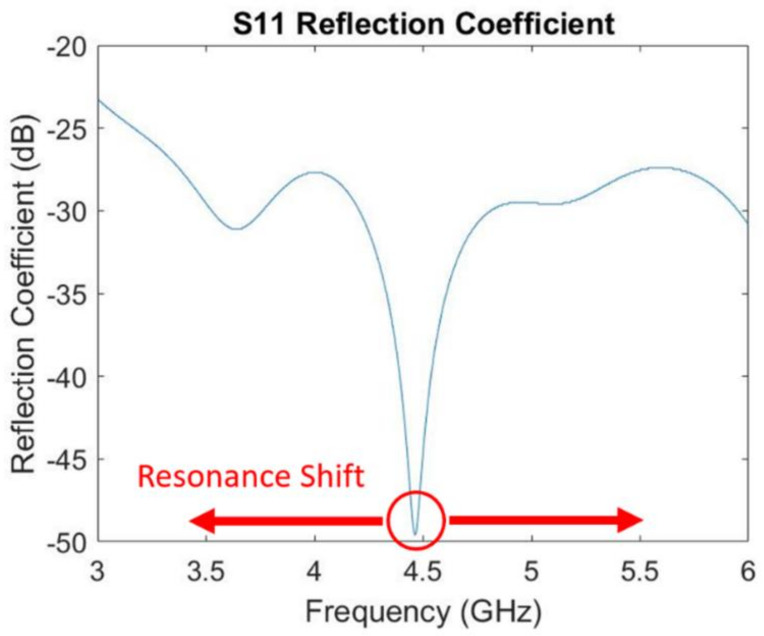
Resonant frequency at the minimum of the reflection coefficient (S11) curve.

The resonant absorption frequency can be seen by applying a frequency sweep and taking the reflection coefficient measurement of the sensor. As the temperature and pressure on the sensor increase, the frequency shifts due to changes in the dielectric constant. The resonance is denoted by a characteristic drop in the reflection coefficient, and the frequency is taken as the minimum of the curve (seen in Figure 15).

### 5.1. Experimental Results on Wireless Temperature Sensing

**Figure 16:**
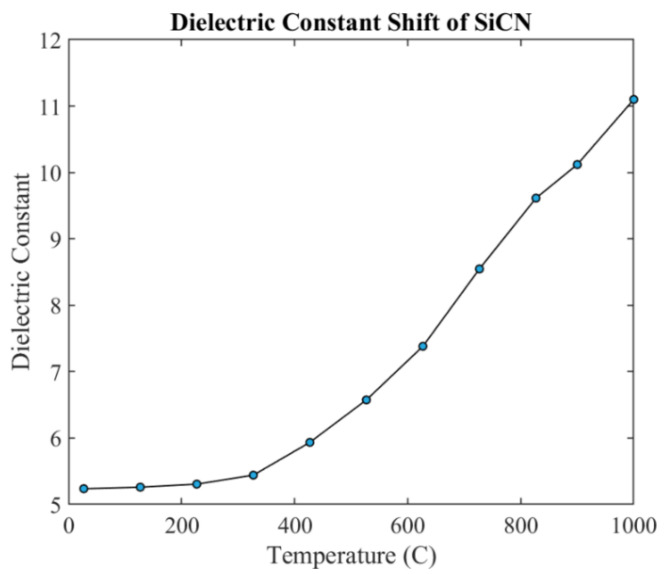
The measurement result of SiCN dielectric constant with increasing temperature.

The measured relative dielectric constant of PDC-SiCN with increasing temperature is shown in Figure 16. The dielectric constant increases with the increase in temperature thus indicating a decrease in the resonance frequency.

**Figure 17:**
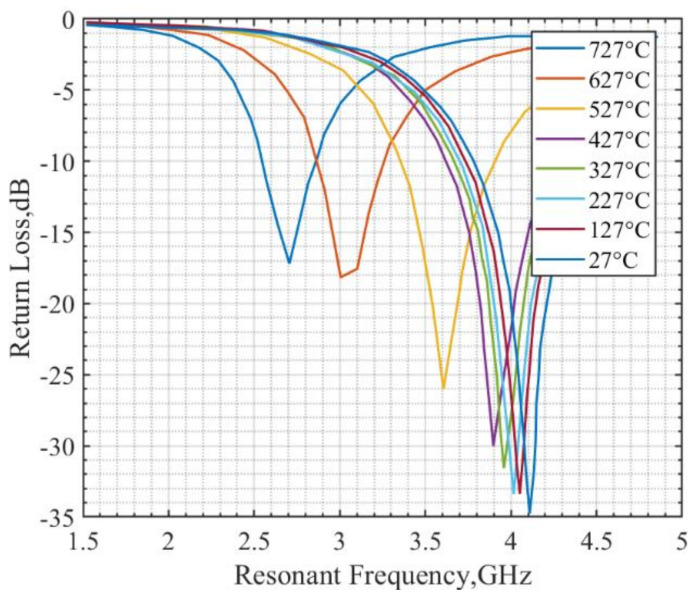
The simulated resonant frequency of temperature sensor changes with temperature.

The patch antenna sensor performance is validated using a three-dimensional (3D) electromagnetic simulation tool. High-Frequency Structural Simulator (HFSS) with dielectric constant data are shown in Figure 16. The S_11_ curves having the lowest return loss at different frequencies are shown in Figure 17. The trough at each temperature is shifted to the left as temperature increases, which means its resonant frequency decreases. With the increase in temperature and pressure, the dielectric constant increases. The resonant frequency is decreasing with this increase in the dielectric constant. In the simulation, a wired micro-patch antenna is used to obtain better S parameters. For different frequencies, the transmission line has different impedance matching degrees, which will introduce different Return Loss (RL) amplitudes.

**Figure 18:**
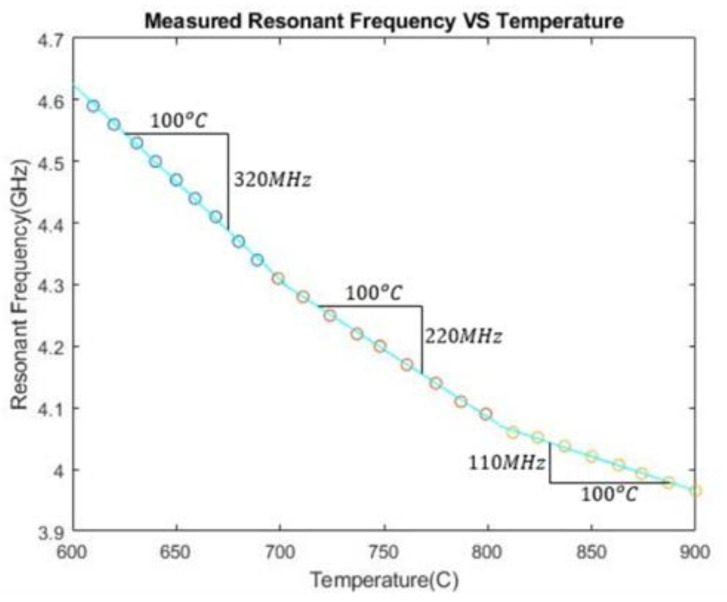
The measured resonant frequency of the PDC-SiCN patch temperature sensor at various ambient temperatures.

According to the simulation result above, the resonant frequency changes with varying temperatures. Next, a PDC temperature sensor is fabricated and measured by experiment. The measured resonance frequency of the sensor for temperatures from 600 °C to 900 °C is plotted in Figure 18. When the temperature increased, the measured resonant frequency decreased from 4.60 GHz to 3.97 GHz. Between different temperature ranges, the resonant frequency decreases at different rates. Overall, the absolute sensitivity is 2.2 MHz/°C.

To verify the accuracy of the temperature sensor, the difference between thermocouple temperature and temperature shown by the temperature sensor can be seen in Table 1. The average value of the absolute mean differences between thermocouple and patch sensor measurements over the entire temperature span is 2.63 °C. Each cycle was run for 10 h, and the experiment was repeated for six cycles.

### 5.2. Experimental Results on the Wireless Pressure Sensing

**Figure 19:**
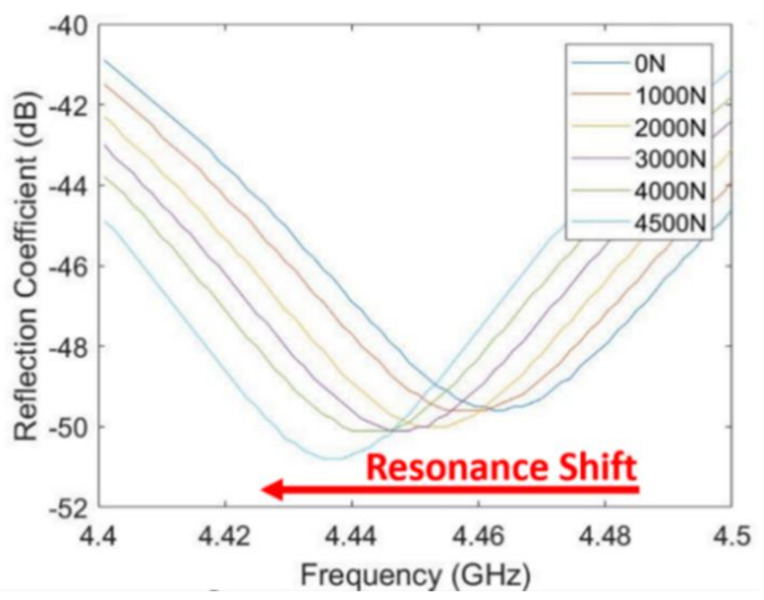
The resonant frequency of pressure sensor changes with varying pressure.

The resonance frequency for force from 0 N to 4500 N is measured and plotted in Figure 19. When the pressure increases, the measured resonant frequency was decreased from 4.455 GHz to 4.435 GHz. These results indicate that the resonant frequency of the sensors with fixed dimensions is pressure-dependent.

**Figure 20:**
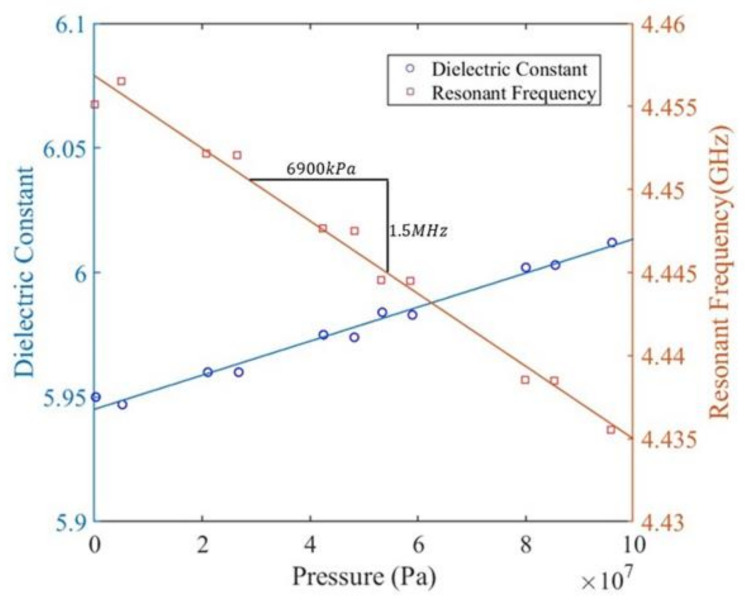
Ramp up Pressure vs. Frequency of pressure sensor during the experiment.

The pressure was determined using the area of the sensor over which the load was applied as well as the force applied using the load cell. The measured dielectric constant of PDC-SiCN and resonant frequency of sensor change with the increasing pressure are shown in Figure 20. From 0 Pa to 9.51 × 10^7^ Pa, a roughly 20 MHz shift in resonance can be seen in the figure.

The S-parameter describes the input-output relationships between ports in an electrical system. The S_11_ response illustrates transmission through port 1 and reception from port 1, which is often referred to as the reflection coefficient (RC) as shown in (15) [22].

(15) $$\mathrm{RC}=10\log_{10}\left(\frac{P_{r}}{P_{I}}\right)$$

where *P_I_* is the incident power and *P_r_* is the reflected power. The reflection coefficient represents how much incident power is reflected. The 0 dB RC means that all signals are reflected from the patch antenna sensor so that there is no electromagnetic energy introduced into the sensor. Similarly, a −10 dB RC means that more than 90% of the energy transmitted is introduced into the sensor. When the reflection coefficient is smaller than −10 dB, it is viewed as sufficient energy transfer into the sensor. Based on these facts and in accordance with Figure 16 and Figure 18, enough energy is transferred into the patch sensor at its resonance in all ranges of temperature and pressure.

## 6. Summary and Conclusions

In this paper, wireless patch antenna sensors have been successfully fabricated which demonstrate the capability to measure temperature and pressure at a sensing distance of 0.5 m. The sensors are entirely wireless and do not require a power source. The wireless sensing mechanism is based on the monotonic increase of the dielectric constant of the PDC-SiCN sensing material with respect to an increasing ambient temperature and applied pressure which induces a decrease in the resonant frequency of the sensor. The frequency shift of 0.67 GHz over temperature ranges from 600 °C to 900 °C is observed with an absolute sensitivity of 2.2 MHz/°C. A shift of 0.02 GHz over a pressure range from 0 Pa to 9.51 × 10^7^ Pa is observed with an absolute sensitivity of 0.217 Hz/Pa. The sensor operation is repeatable in the full range of temperature and pressure variation.

This work demonstrates the feasibility of measuring high temperatures and pressures using patch antenna wireless passive sensors in harsh environments. It is anticipated that the sensor could be tailored to reduce the size and increase the sensitivity by altering the material dielectric properties (e.g., dielectric constant). The sensing distance can also be optimized specifically to different environments (high reflection, low reflection). Overall, it is inexpensive, efficient, and aims to reduce maintenance costs. Further, when a wireless sensor network using multiple sensors is formed, pressure and temperature distribution can be measured over a span of sensing area, which means not only the pressure and temperature can be measured simultaneously, but also temperature and pressure distribution can be directly plotted using standard computer software.

What is more, it is envisioned that other printed component technologies such as capacitors, antennas, and traces will be able to be designed and eventually seamlessly printed on a single machine for benefits such as weight reduction, cost savings for low production quantities of tailorable weapon systems to name a few. Both monitoring and optimizing, if possible, temperature and pressure in extreme environments would be broadly useful for many extreme applications to improve efficiency and safety.

## Figures and Tables

**Table 1:** A comparison of temperature measurement by patch antenna sensor and thermocouple.

Temperature Range	600–650	650–700	700–750	750–800	800–850	850–900
|∆e| (°C)	0.78	6.56	2.93	0.31	1.03	6.15

|∆e*| = |(thermocouple measurement) − (patch antenna sensor measurement)|.

## Nomenclature of Variables

*τ*(*T*)	Temperature-dependent polarization relaxation time
*T*	Temperature
*τ*_0_	Relaxation time pre-factor
*E_a_*	Activation energy
*R*	Gas constant
*ε*′	Real part of dielectric constant
*ε_s_*	Static dielectric constant
*ε_α_*	Relative dielectric constant at the high-frequency limit
*ω*	Angular frequency
*ε*	Dielectric constant
*p*	Applied pressure
M	Atomic weight in grams
*ρ*	Material density
N	Avogadro’s number
*α*	Atomic polarizability
*P_t_*	Power fed into the transmitting antenna
*P_r_*	Power available at the receiver
*A_t_*	Effective area of the transmitting antenna
*A_r_*	Effective area of the receiving antenna
*d*	Distance between antennas
*λ*	Microwave’s wavelength
*R*	Boundary distance between antenna and sensor
*D*	The largest dimension of the antenna
$\varepsilon^{\prime \prime }$	Imaginary part of the dielectric constant
C	Speed of light
$f_{r}$	Designed resonant frequency
$\varepsilon_{r}$	Relative dielectric constant
*h*	Substrate thickness
$\varepsilon_{reff}$	Effective dielectric constant with fringing field
W	Width of patch
*L*	Length of patch
$L_{eff}$	Effective length of patch
$\lambda_{d}$	Wavelength of designed resonant wave
